# Editorial: Transcriptomics of fruit growth, development and ripening

**DOI:** 10.3389/fpls.2024.1399376

**Published:** 2024-04-05

**Authors:** Neftali Ochoa-Alejo, Maria Carmen Gómez-Jiménez, Octavio Martínez

**Affiliations:** ^1^ Departamento de Ingeniería Genética, Centro de Investigación y de Estudios Avanzados del Instituto Politécnico Nacional, Unidad Irapuato, Irapuato, Guanajuato, Mexico; ^2^ Laboratory of Plant Physiology, Universidad de Extremadura, Badajoz, Spain; ^3^ Unidad de Genómica Avanzada, Centro de Investigación y de Estudios Avanzados del Instituto Politécnico Nacional, Irapuato, Guanajuato, Mexico

**Keywords:** carbamoyltransferases, carotenoids, core genes, flavor, fruit, histone acetyltransferases, metabolomics, regulatory network

## Introduction

Fruits are organs that hold seeds in plant species. From a botanical point of view, fruit is defined as a mature ovary (Roth, 1977), or as a structure developing from the gynoecium of one flower as the result of pollination or parthenocarpy (Bobrov and Romanov, 2019), or, additionally, as the flower in the state of seed germination (Knoll, 1939). An enormous variety of fruits with different forms, sizes, textures, colors, flavors, and aromas exists in nature (Klee, 2010; Rodríguez et al., 2012; Stournaras et al., 2012; Wu et al., 2018; Kapoor et al., 2022). Fruits are classified as fleshy or dry. Fleshy fruits are distributed in nature primarily by animals, whereas dry fruits may be dispersed by animals, wind, or water (Carey et al., 2019). Dry fruits are classified as dehiscent when they release seeds into environment (the seeds are discarded before or after consuming), or indehiscent those that release seeds in protected fruit wall propagules. Fleshy fruits are classified as climacteric (bananas, tomatoes, apricot, pears, mangoes, apricots, peaches, apples, papayas, guava, nectarines, blueberry, plum, passion fruit, cantaloupe, and avocados) or non-climacteric (grapefruit and lemon, berries such as raspberry, strawberry, cherry, grapes, pineapple, melon, watermelon, and pomegranate). In climacteric fruits, a burst of ethylene biosynthesis and an increase in respiration is observed at the onset of ripening. On the other hand, non-climacteric fruits lack the autocatalytic ethylene burst. Fruits are plant organs of nutritional value for animals and humans since they are sources of food, fiber, vitamins, minerals, carbohydrates, organic acids, amino acids, proteins, polyphenols (flavonoids and stilbenes), sterols, fatty acids, lipids, and pigments with antioxidant properties, among others (McKee and Latner, 2000; Zamora et al., 2001; Avila-Sosa et al., 2019; Wu et al., 2019; Golovinskaia and Wang, 2021; Alvi et al., 2022; Kasampalis et al., 2022; Yun et al., 2022; Bures et al., 2023; Cavalcante de Oliveira et al., 2023). Fruits are formed from single ovaries and may or may not involve inclusion of accessory floral tissues like the floral receptacle. After pollination, flowers undergo complex processes involving cell division, cell enlargement, and cell differentiation mediated by the expression of hundreds or even thousands of genes under a fine, harmonic, and sequentially regulated program to promote growth, development, ripening and, finally, senescence (Gillaspy et al., 1993; Karlova et al., 2014). Fruit initiation, growth, development, ripening, and senescence are influenced by genetic, epigenetic, hormonal, and environmental factors (Seymour et al., 2008, 2013; Chen et al., 2022). A powerful approach to study those genes expressed during the growth, development, ripening, and senescence is transcriptomics through RNA-Seq analysis (Wang et al., 2009; Tarazona et al., 2011; Trapnell et al., 2013; Li D, et al., 2022). The set of all RNA molecules transcribed in an organ or tissue at a particular point of time under a given set of environmental conditions constitute the transcriptome (Velculescu et al., 1997; Martínez-López et al., 2014). *Arabidopsis thaliana* and tomato (*Solanum lycopersicum*) have been used as model plants to investigate dry and fleshy fruit biology, respectively (Seymour et al., 2013; Gómez et al., 2014). This Research Topic focuses on transcriptomic research in different plant species revealing changes in gene expression and key regulatory gene networks involved in fruit growth, development, ripening, and senescence.

## Fruit transcriptomics

Transcriptional changes in fleshy fruits during growth, development, and ripening were previously reviewed (Karlova et al., 2014); transcriptomic analysis of the dry fruit (silique) of the model plant *Arabidopsis thaliana* was published by Mizzotti et al. (2018). In general, fruit formation, development, ripening, and senescence involve metabolic changes regulated by hormones and environmental factors; these changes include variations in metabolites, color, flavor, and aroma, and the softening process in the case of fleshy fruits. All these changes are usually regulated at the transcriptional level by diverse transcription factors (TFs). Transcriptional studies on fruit growth, development, ripening, and senescence have been published by different authors for several tropical plant species, including mango (*Mangifera indica* L.) (Pandit et al., 2010), pineapple (*Ananas comosus*) (Koia et al., 2012), melon (*Cucumis melo* L.) (Saladié et al., 2015), watermelon [*Citrullus lanatus* (Thunb.) Matsun. & Nakai)] (Zhu et al., 2017), litchi (*Litchi chinensis* Sonn.) (Liu et al., 2017), citrus (*Citrus sinensis*) (Feng et al., 2019), and goji berry (*Lycium barbarum*) (Zhao et al., 2020), or on temperate species such as almond (*Prunus dulcis*) (Guo et al., 2021), apple (*Malus domestica*) (Xu et al., 2020; Li M, et al., 2022), peach (*Prunus persica*) (Li M, et al., 2022), pear (*Pyrus* spp.) (Xie et al., 2013; Zhang et al., 2016), blueberry (*Vaccinia* spp.) (Gao et al., 2021; Yang L, et al., 2021), grape berry (*Vitis vinifera*) (Minio et al., 2019), and raspberry (*Rubus idaeus*) and strawberry (*Fragaria vesca*) (Zhou et al., 2023), have been reported. Transcriptomics of fruits used as vegetables, such as cucumber (*Cucumber sativus*), and chili pepper (*Capsicum* spp.), have been also published (Ando et al., 2012; Martínez et al., 2021, respectively). As an example of transcriptomic analysis, Galla et al. (2009) described a computational annotation of differentially expressed genes in fruits of olive (*Olea europea* L. cv. Leccino), a non-climacteric species, at three developmental stages (initial fruit set, completed pit hardening, and veraison) using subtractive hybridization libraries; 1,132 clones were sequenced, 60% of which presented similarity with known proteins, and were annotated by Gene Ontology (GO). Bioinformatic analysis revealed a significantly different distribution of the annotated GO category. The olive fruit-specific transcriptome dataset was used to query all known KEGG (Kyoto Encyclopedia of Genes and Genomes) metabolic pathways for characterizing and positioning retrieved EST (Expressed Sequence Tags) records, finding a predominance of KEGS maps associated to carbohydrate (enzymes involved in starch and sucrose metabolism, glycolysis and gluconeogenesis), fatty acid (fatty acid biosynthesis and lipid degradation), and secondary metabolism (phenylpropanoids, terpenoids, flavonoids, alkaloids, and caffeine biosynthesis, and limonene and pinene degradation). Moreover, genes involved in amino acid biosynthesis and metabolism were also differentially expressed. Genes related to hormone biosynthesis and action were also found to be differentially expressed. Auxin responsive transcription factor genes, such as *ARF1* and *ARF7*, were up- and down-regulated, respectively. Biosynthesis of abscisic acid (ABA) was stimulated as well as the expression of the ABA-biosynthesis related enzyme genes *ABA2* (*ABA DEFICIENT 2*), and *AAO3* (*ABSCISIC ALDEHYDE OXIDASE*). Genes related to the biosynthesis and action of indoleacetic acid (IAA), ABA, gibberellic acid (GA), ethylene, cytokinins, jasmonate, and salicylic acid were differently regulated according to the type of hormone. Genes encoding transcription factors were mainly down-regulated throughout fruit development, while some others were related to protein modification and degradation. In a further study (Parra et al., 2013), a comparative transcriptional profiling analysis of pericarp and the abscission zone (AZ) tissues in olive ripe-fruit from cv. Picual was conducted, and it was found that 4,391 genes were differentially expressed (DEG); in general, AZ tissue exhibited higher response to external stimuli than did ripe fruit, with higher expression of auxin-signaling genes, lignin catabolic and biosynthetic pathway, and of biosynthetic pathways of aromatic amino acid, isoprenoids, protein amino acid dephosphorylation, photosynthesis, and amino acid transport. Ripe fruit showed an enrichment in transcripts involved in ATP synthesis coupled proton transport, glycolysis, and cell-wall organization. Additionally, approximately 150 transcripts encoding putative TFs of diverse families were identified (37 fruit TFs and 113 AZ TFs); the most abundant TFs in ripe fruit were MADS-box proteins (TAGL2, AGL9, AG1), homeobox domain proteins, zinc finger proteins (ZF), basic helix-loop-helix proteins (bHLH), and basic leucine zipper proteins (bZIP). Among the 37 TF genes, 25 were exclusively expressed in fruit [6 *ZF*, 5 homeobox proteins, 5 *bHLH* domain class, 3 *bZIP*, 1 *MADS*-box (*AG1*), 1 *MYB* (*MYBA22*), 1 *NAC*, 1 *Aux/IAA* (*IAA1*), 1 *CAMTA*, and 1 *C2H21*. In the AZ tissue *ZF*, *bHLH*, and *bZIP* genes were highly expressed, but at different proportions, and one member of the *E2F* family and 9 *WRKY* TFs genes were exclusively expressed.

In some cases, transcriptional studies have been focused on just early or late stages of fruit development and ripening (Ando et al., 2012; Gómez et al., 2014; Liu et al., 2017), or on a specific tissue (Parra et al., 2013; Tafolla-Arellano et al., 2017; Luo et al., 2018; Zhang et al., 2021; Zhou et al., 2022), or even on cell transcriptomics (Martin et al., 2016; Shinozaki et al., 2018). Moreover, transcriptomics of hormones-related gene expression (Zhu et al., 2011; Huang et al., 2014; Van de Poel et al., 2014; Tang et al., 2015; Briegas et al., 2020; Kou et al., 2021; Qiao et al., 2021; Camarero et al., 2023), or the effects of environmental/stress factors on fruit gene expression (Li et al., 2019; Cramer et al., 2020; Waite et al., 2023), biosynthesis/metabolism genes expressed during fruit development (Yu et al., 2021; Zhang et al., 2021; Diao et al., 2023), transcriptomic changes due to the evolution/domestication processes (Martínez et al., 2021; Borredá et al., 2022; Gramzou et al., 2022), expression of transcription factors genes and gene networks (Ye et al., 2015; Villa-Rivera et al., 2022), transcriptomics of fruit quality (Yang H, et al., 2021; Lei et al., 2022), fruit shape (Tsaballa et al., 2011; Shi et al., 2023) and size-related genes (Huang et al., 2023; Liu et al., 2023), or postharvest transcriptomic changes (Wang et al., 2018; Romero et al., 2022), have been documented as an approach to reveal the mechanisms and factors involved in the growth, development, ripening, and senescence of fruits.

## Articles and insights

This Research Topic is composed of nine articles, and among them, two deal with different aspects of fruit transcriptomics, including growth, development, and ripening (Gaete-Eastman et al.; Rajewski et al.), and in one case the transcriptomic analysis was combined with metabolomics to study fruit flavor (Lu et al.). Basically, Rajewski et al. looked for differentially expressed genes in pericarp tissues of dry fruits from *Nicotiana obtusifolia* and *Solanum pimpinellifolium*, two wild species, and of the fleshy fruits from *Solanum lycopersicum* and *Cucumis melo*, two climacteric species, and, interestingly, they found core genes (121) during fruit development and ripening when *Arabidopsis thaliana* fruits (dry) were also included in the analysis; on the other hand, in the comparative gene expression profiles between the wild tomato (*S. pimpinellifolium*) and the domesticated species (*S. lycopersicum*), 1,472 genes showed divergent expression patterns and there were Gene Ontology enrichments for plant-type cell wall organization and lipid biosynthetic processes. Furthermore, expression analysis of ethylene, pigment, and flavor biosynthesis-related genes exhibited statistically significant differences between cultivated and wild tomato. Additionally, fruit size-, firmness-, and lignification-related transcription factors differed in expression between wild and domesticated tomato. This study revealed insights on the effects of domestication on gene expression profiles and on the evolutionary process in dry and fleshy fruits. In another article, Gaete-Eastman et al. reported an RNA-Seq transcriptomic analysis across different developmental stages and ripening of the non-climacteric fruits of *Fragaria chiloensis*, a Chilean strawberry species, and the main findings refer to the differential expression of ABA biosynthesis-related genes involved in softening, color, and aroma production, which are regulated by transcription factors such as FcMYB1. In an integrated metabolomic and transcriptomic study of jujube (*Ziziphus jujuba*) fruits during development and maturation, Lu et al., described differentially expressed transcription factor genes highly correlated with sugars and organic acids accumulation, important compounds involved in the fruit flavor.

Two articles were focused on the expression analysis of specific gene families during fruit development and ripening (Cai et al.; Dhar et al.). A genome-wide analysis of histone acetyltransferase (HAT) and histone deacetylase (HDAC) gene families and their expression in chili pepper (*Capsicum annuum* L.) fruits during development and ripening revealed a total of 30 HAT and 15 HDAC, which were differentially expressed and may be involved in the regulation of fruit development- and ripening-related phytohormone metabolism and signaling through changes in chromatin acetylation/deacetylation activities (Cai et al.). In the other case, Dhar et al. reported a genome and transcriptome-wide analysis of carbamoyltransferase genes (play roles by regulating the urea cycle, *de novo* pyrimidine biosynthesis, and arginine biosynthesis in prokaryotes and eucaryotes) in major fleshy fruits (30) from an evolutionary point of view, and they found 393 carbamoyltransferase genes conserved in the plant kingdom, indicating a fundamental biological relevance.

Additionally, the overexpression of the carotenoid biosynthesis-related structural gene *PSY1* as an approach to increase the carotenoid content in the skin and flesh of apple (*Malus domestica*) fruits was investigated, and an increase in carotenoid content (β-carotene being the most accumulated) was observed (Ampomah-Dwamena et al.).

A postharvest molecular study on the climacteric apple (*Malus domestica* cv. Golden Delicious) fruit quality after wax coating treatment was reported here, and the main findings were the inhibition of the expression of ethylene biosynthesis, chlorophyl degradation, and carotenoid biosynthesis-related genes (Si et al.). In one article, key genes associated with seed germination dormancy as affected by cold stratification in *Fritillaria taipaiensis* P.Y.Li (a traditional Chinese medicinal plant) were analyzed and they found that stratification at 4 °C induced an up-regulation of genes involved in gibberellic acid and auxin biosynthesis (Yang et al.). Finally, a comparative transcriptomic analysis between plants bearing prickles and non-prickled plants revealed the possible developmental mechanism of prickle formation in the important forest species *Zanthoxylum bungeanum* (Su et al.).

In conclusion, this Research Topic included not only interesting articles on transcriptomics covering different aspects of growth, development, and ripening of fruits from different plant species, but involving even transcriptomic changes occurring during postharvest conditions and germination of seeds, which certainly will be of motivation for those researchers working on this important biological process.

## Author contributions

NO-A: Conceptualization, Funding acquisition, Investigation, Visualization, Writing – original draft, Writing – review & editing. MG-J: Writing – review & editing. OM: Writing – review & editing.
